# Studies of Indium Tin Oxide-Based Sensing Electrodes in Potentiometric Zirconia Solid Electrolyte Gas Sensors

**DOI:** 10.3390/s21072345

**Published:** 2021-03-27

**Authors:** Stefan Dietrich, Mihails Kusnezoff, Alexander Michaelis

**Affiliations:** 1 Fraunhofer Institute for Ceramic Technologies and Systems IKTS, Winterbergstr. 28, 01277 Dresden, Germany; mihails.kusnezoff@ikts.fraunhofer.de; 2 Institute of Materials Science, Technische Universität Dresden, 01069 Dresden, Germany; alexander.michaelis@ikts.fraunhofer.de

**Keywords:** potentiometric solid electrolyte sensor, mixed potential, indium tin oxide, internal reference gas sensor

## Abstract

A zirconia-based potentiometric solid electrolyte gas sensor with internal solid state reference was used to study the response behavior of platinum cermet and indium tin oxide sensing electrodes. Target gases included both oxygen and carbon monoxide in nitrogen-based sample gas mixtures. It was found that with the indium tin oxide sensing electrode, the low-temperature behavior is mainly a result of incomplete equilibration due to contaminations of the electrode surface. On the other hand, some of these contaminant species have been identified as being pivotal for the higher carbon monoxide sensitivity of the indium tin oxide sensing electrode as compared to platinum cermet electrodes.

## 1. Introduction and Theoretical Background

For more than four decades, potentiometric gas sensors based on zirconia solid electrolytes have been widely employed for the determination of oxygen content and air-to-fuel ratio in automotive applications and industrial processes. This type of sensor usually features two electrodes, deposited on and electrochemically coupled by the oxygen ion conducting solid electrolyte. Historically, yet still state-of-the-art in many applications, platinum (Pt) or Pt-based cermets are used as electrodes while zirconia serves as solid electrolyte. Typically, the ionic conductivity of zirconia is enhanced by adding metal oxides, like yttria (Y_2_O_3_) or scandia (Sc_2_O_3_) and ceria (CeO_2_). Prominent examples of these so-called stabilized zirconia (SZ) are 8YSZ and 10Sc1CeSZ, where the numbers represent the mole fractions of the dopant oxides, respectively.

### 1.1. Sensor Principle and Response to Oxygen

Schematically, this type of potentiometric solid electrolyte gas sensor can be described by the concentration cell

(1) $$\left(p_{O_{2}}^{\prime }\right)\;\mathrm{Pt}\;\left|\;\mathrm{SZ}\;\right|\;\mathrm{Pt}\;\left(p_{O_{2}}^{\prime \prime }\right)\;,$$

where $p_{O_{2}}^{\prime }$ und $p_{O_{2}}^{\prime \prime }$ represent the oxygen partial pressures at the platinum electrodes. Depending on the sensor design, the electrodes can be exposed to either the same or dissimilar gas environments.

In the simplest case of exposure to a mixture of an inert gas like nitrogen and oxygen, adsorption leads to an oxygen partial pressure dependent coverage of chemisorbed oxygen species on the electrode surface. When the sensor is operated at a sufficiently high temperature of 350–950 °C, a thermodynamic equilibrium is established between oxygen, electrons and mobile oxygen ions at the so-called triple phase boundary (TPB) between gas phase, electrode and solid electrolyte, respectively [1,2]:

(2) $$O_{2}\left(g\right)+4e^{-}\left(\mathrm{Pt}\right)⇌2O^{2-}\left(\mathrm{SZ}\right)\;.$$

In this case, the oxygen chemical potentials $\mu_{O_{2}}^{\prime }$ and $\mu_{O_{2}}^{\prime \prime }$ at the electrodes are given by

(3) $$\mu_{O_{2}}^{\prime }=\mu_{O_{2}}^{\prime 0}+RT\ln p_{O_{2}}^{\prime },$$

(4) $$\mu_{O_{2}}^{\prime \prime }=\mu_{O_{2}}^{\prime \prime 0}+RT\ln p_{O_{2}}^{\prime \prime }\;,$$

where *R* is the universal gas constant, *T* the absolute temperature, and $\mu_{O_{2}}^{\prime 0}$ and $\mu_{O_{2}}^{\prime \prime 0}$ are the standard chemical potentials, respectively [3]. If the oxygen partial pressures and thus the chemical potentials differ on either side of the solid electrolyte, an electric potential difference (also termed electromotive force, emf),

(5) $$\Delta V=\frac{1}{4F}\int_{\mu_{O_{2}}^{\prime }}^{\mu_{O_{2}}^{\prime \prime }}\;t_{\mathrm{ion}}\;\cdot \;d\mu_{O_{2}},\;$$

is generated between the electrodes [4]. Here, *F* is the Faraday constant and $t_{\mathrm{ion}}\;=\;\sigma_{ion}/\sigma_{tot}$ is the ionic transference number of the solid electrolyte, which describes the ratio between ionic ($\sigma_{ion}$) and total conductivity ($\sigma_{tot}$). To simplify Equation (5), the electronic contribution to the solid electrolyte’s total conductivity is assumed to be negligible so that $t_{\mathrm{ion}}\approx 1$. In the case of YSZ and 10Sc1CeSZ, this usually holds for operation in non-reducing gas mixtures. For example, Shimonosono et al. have found an electronic conductivity of 2 × 10^−7^–5 × 10^−6^ S cm^−1^ for 10Sc1CeSZ at 900 °C in a wide range of oxygen partial pressures of 1 × 10^−15^–1 × 10^−2^ bar [5]. Under this assumption, Equation (5) yields the open circuit voltage

(6) $$U_{Nernst}=\Delta V=\frac{RT}{4F}\ln\frac{p_{O_{2}}^{\prime \prime }}{p_{O_{2}}^{\prime }}\;,$$

that can be measured between the electrodes. This so-called *Nernst voltage* is a measure of the oxygen partial pressure difference between both electrodes [2,4,6,7].

### 1.2. Reference Electrode

For reproducible determination of the oxygen partial pressure in a sample gas, one electrode is required to provide a sufficiently stable reference potential; hence, the electrodes are commonly referred to as sensing (SE) and reference electrode (RE). Various concepts are available to facilitate such reference potential, some of which are explained elsewhere [8]. For the work described in the present article, a so-called solid state reference electrode has been used. This type of reference features a mixture of a metal and its oxide which establishes a temperature-dependent oxygen partial pressure. The function of such reference is based on the Gibbs phase rule, according to which the oxygen partial pressure in a three-phase mixture of a metal (M), its oxide, and oxygen is constant at a given temperature. For a reaction represented by

(7) $$xM+yO_{2}⇌M_{x}O_{2y},$$

the enthalpy of formation under standard conditions, $\Delta G^{0}$, is given by

(8) $$\Delta G^{0}=yRT\;\cdot \;\ln\;p_{O_{2}}\;$$

according to refs. [2,9]. Here, *x* and *y* are stoichiometric coefficients, and it is assumed that the activity of solid reactants is unity. Consequently, the oxygen partial pressure $p_{O_{2}}^{ref}$ defined by such metal-metal oxide reference can be calculated:

(9) $$p_{O_{2}}^{ref}=\exp\frac{\Delta G^{0}}{yRT}\;.$$

In literature, various reference systems based on different metal-metal oxide couples have been described [2,10,11]. Although the implementation of such reference requires gas-tight sealing to prevent unwanted oxidation of the metal component, it will be quite resilient towards small oxygen leakages in the sensor. Long-term stability of this kind of reference depends on a multitude of factors, like materials used, initial phase composition, operating conditions, and possible leakages, and may range from a few minutes to several years.

### 1.3. Sensor Response in the Presence of a Reducing Gas

If, in a sample gas consisting of nitrogen and oxygen, an additional reducing gas like carbon monoxide (CO) is present at the sensing electrode, the relevant half cell can be described by

(10) $$(p_{O_{2}},\;p_{\mathrm{CO}})\;\mathrm{SE}\;|\;\mathrm{SZ}\;,$$

where $p_{\mathrm{CO}}$ is the CO partial pressure in the gas mixture. In this case, various processes, which are dependent on electrode material and morphology, operating conditions and the ratio of oxygen and carbon monoxide partial pressures, contribute to the potential and thus, the sensor voltage. Usually, two main reactions of carbon monoxide are relevant for sensor response: the oxidation of CO with oxygen adsorbed at the electrode surface described by the simplified equation

(11) $$\mathrm{CO}\left(g\right)+O_{\mathrm{ads}}\left(\mathrm{Pt}\right)\to {\mathrm{CO}}_{2}$$

and the electrochemical interaction with lattice oxygen of the solid electrolyte at the triple phase boundary

(12) $${\mathrm{CO}}_{\mathrm{ads}}\left(\mathrm{TPB}\right)+O_{O}^{\times }\left(\mathrm{SZ}\right)⇌{\mathrm{CO}}_{2}\left(g\right)+V_{O}^{⦁⦁}\left(\mathrm{SZ}\right)+2e^{\prime }\left(\mathrm{SZ}\right)\;.$$

In these equations, the subscript ads refers to an adsorbed species, while $O_{O}^{\times }$ denotes the host oxygen ion, $V_{O}^{⦁⦁}$ a doubly positive charged oxygen vacancy and $e^{\prime }$ an electron. The chemical reaction according to Equation (11) results in CO being oxidized to carbon dioxide (CO_2_), which effectively leads to a decrease in $p_{\mathrm{CO}}$ and a reduced oxygen coverage at the electrode surface. As a result, less oxygen is available for potential generation at the triple phase boundary according to Equation (6). If an electrode material with high catalytic activity towards CO oxidation is used in conjunction with sufficient oxygen, carbon monoxide may almost entirely be converted to CO_2_ before reaching the triple phase boundary. If the operating temperature is high enough, the result is an equilibrated gas mixture and the measured sensor voltage corresponds to the resulting equilibrium oxygen partial pressure.

If the gas is not equilibrated, for example, due to an electrode with low catalytic activity or insufficient thermal activation, a fraction of the CO reaches the triple phase boundary and is oxidized electrochemically via the reaction given in Equation (12). Under these conditions, a steady state develops, where the electrochemical oxygen reduction (Equation (2)) and the electrochemical CO oxidation (Equation (12)) proceed at the same rate, leading to the generation of a so-called mixed potential [12,13,14,15,16]. In most scenarios, a certain amount of chemical CO conversion at the electrode surface cannot be ruled out and the resulting mixed potential is a function of the reduced effective partial pressures of O_2_ and CO. Mixed potential generation is therefore mainly controlled by the partial pressures of the incident gases and the electrode kinetics, which in turn are influenced by the operating temperature, electrode materials, and morphology [15].

### 1.4. Electrode Materials and Aim of the Present Studies

The mechanisms described in the previous section have been understood quite well for standard platinum-based sensing electrodes. Due to the high catalytic activity of the material, platinum electrodes serve well to determine the equilibrium oxygen partial pressure. If the aim of the measurement is the detection of an oxidizable gas like CO in gas mixtures containing oxygen, avoiding equilibration is paramount. To achieve this, additional electrode coatings that restrict oxygen adsorption or electrodes composed of catalytically less active materials, like gold, silver, and selected metal oxides, may be used. Although the gas sensing properties of such materials have been investigated by several researchers in the past as well, particularly for metal oxide electrodes, no comprehensive picture for the fundamental interaction of gas species with the electrodes is available yet [17,18,19,20]. A prominent material which has seen significant research for application in gas sensing during the past decades is indium tin oxide (ITO). ITO is an n-type semiconducting metal oxide composed of indium(III) oxide (In_2_O_3_) and tin (IV) oxide (SnO_2_) with a typical mass ratio of 90:10. In gas sensing, ITO has mostly been investigated as chemoresistive thin film to detect a wide range of gases, like CO, CO_2_, CH_4_, H_2_, NO_x_, NH_3_, benzene, various alcohols, and further hydrocarbons [21,22,23,24,25,26,27,28,29,30,31,32,33,34,35,36]. At the same time, the use as screen printed thick film has only been described by very few authors [24,32,37]. Studies of ITO-based sensing electrodes in electrochemical solid electrolyte gas sensors have so far only been reported by two research collaborations [38,39].

In a previous article, the results of combined adsorption/desorption and electrical conductivity studies on the interaction of ITO powders and films with various gas species have been described [40]. In the present paper, these results are used to explain the gas sensing behavior of a potentiometric zirconia-based solid electrolyte gas sensor with ITO sensing electrode exposed to CO in oxygen rich gas environment. The findings are compared to results obtained with the same type of sensor equipped with a standard platinum-based sensing electrode. Results of additional adsorption/desorption studies on Pt which are used to explain the experimental data have been reported in a previous publication [8].

## 2. Materials and Methods

**Figure 1:**
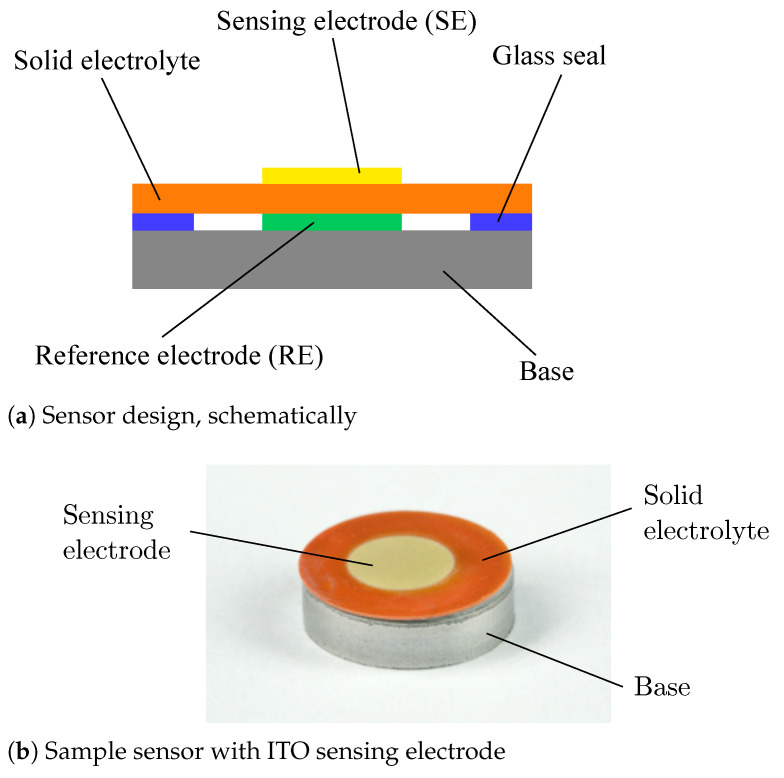
Potentiometric zirconia solid electrolyte sensor with internal reference.Potentiometric zirconia solid electrolyte sensor with internal reference

To allow facile characterization of sensing electrode materials, a potentiometric zirconia solid electrolyte sensor with internal reference has been developed. The sensor comprises of two main components: a disk shaped metal base of Crofer 22 APU high temperature steel (ThyssenKrupp VDM GmbH, Werdohl, Germany) with a thickness of 2.5 mm and a diameter of 10 mm and an electrochemical sensing cell (Figure 1). The sensing cell is an asymmetric cell based on a 10Sc1CeSZ solid electrolyte disk with a thickness of 165 µm and a diameter of 10 mm. Every cell is equipped on one face with a circular reference electrode of 5 mm diameter deposited from a NiO/8YSZ cermet paste and which was fired in a box-type furnace at 1225 °C in air. On the other face, a sensing electrode of the same size was deposited from either a Pt/10Sc1CeSZ cermet paste or an ITO paste. Details of pastes, as well as metal and metal oxide powders, are described in a previous article [40]. While the Pt cermet electrodes were also fired at 1225 °C in air, ITO electrodes have been deposited and fired in a separate step after joining of the sensor, to avoid ITO exposure to high temperature vacuum conditions.

For sensor joining, a ring made of aluminium borosilicate glass green tape with inner and outer diameters of 6 and 10 mm, respectively, was deposited on one face of the Crofer base. The glass had previously been developed for joining YSZ and Crofer 22 APU in high temperature solid oxide fuel cells and was tested to be suitable for the present application [41]. The glass was pre-treated in a box-type furnace at 800 °C in air. For sensor assembly, the sensing cell is placed on the pre-treated Crofer base so that the NiO reference electrode faces the Crofer base. Joining of both components has been performed in a vacuum furnace (LHTM 200-300/16-1G, Carbolite Gero GmbH & Co. KG, Neuhausen, Germany) at an absolute pressure of $1\times 10^{-5}$ mbar and a temperature of 1000 °C. Due to the shrinkage of the glass during the high temperature joining process, the sensing cell settles onto the Crofer base, facilitating mechanical and electrical contact between reference electrode and the base. At the same time, vacuum conditions allow the reduction of the NiO reference electrode and help to establish a low oxygen partial pressure inside the sealed gas tight sensor volume. After joining, the ITO thick film paste was deposited by either mask or screen printing on those sensors without previously applied sensing electrode. The resulting ITO thick films have then been fired for 1 h at 700 °C in air. This temperature had been obtained from previous studies, where it lead to a good compromise between film adhesion on the solid electrolyte, a sufficiently porous structure and a high electrical conductivity.

For sensor characterization, samples were placed onto an alumina sample holder which was then inserted into a quartz glass tube reactor housed inside a tube furnace. The reactor inlet was connected to a gas supply line providing dry N_2_, O_2_, and CO as required, while the outlet was connected to an exhaust line. Two methods have been used to adjust composition of the feed gas: mass flow controllers (Bronkhorst High-Tech B.V., Ruurlo, The Netherlands) served to dose N_2_, O_2_ concentrations larger than 1000 ppm and all CO concentration levels. In addition, in the gas line immediately upstream and downstream of the reactor inlet and outlet, respectively, an electrochemical electrolysis device (SGM5-EL, Zirox Sensoren und Elektronik GmbH, Greifswald, Germany) was placed, hereinafter referred to as Cell 1 and Cell 2. Both Cell 1 and Cell 2 served to measure oxygen partial pressure in the feed and exhaust gas; in addition, Cell 1 was used to dose oxygen concentrations of up to 1000 ppm into the feed stream. To avoid unwanted CO conversion in the feed gas before reaching the sample sensor, Cell 1 was disabled in all measurements where CO was involved. Electrical connections to the sensor were made using four Au wires routed through the sample holder. To ensure reliable electrical contact, the wire pairs were fixed at sensor base and sensing electrode using a small amount of Au conductor paste, which was then fired at 700 °C in synthetic air. A type 2400 source meter (Keithley Instruments/Tektronix, Inc., Beaverton, OR, USA) was used to measure sensor voltage via 4-terminal sensing. In addition, a type-S thermocouple was placed on the sample holder in close proximity of the sensor and read out using an Almemo 2390-3 data logger unit to obtain precise temperature information during measurement (Ahlborn Mess- und Regelungstechnik, Holzkirchen, Germany). Sensor operating temperature was provided by the tube furnace, which was connected to a control unit equipped with temperature controller (HTM Reetz GmbH, Berlin, Germany; ET-2416, Schneider Electric Systems/Eurotherm, Limburg an der Lahn, Germany).

## 3. Results and Discussion

The complete characterization procedure described in this section has been performed with one sensor of each sensing electrode type. Every type of measurement was repeated several times over the course of 2–4 weeks. Due to the minuscule deviations between individual runs, no error bars have been included in the data. Additional sensors have been used to supplement measurement results, the data were in good agreement. Unless stated otherwise, reported sensor voltages are versus the Ni/NiO reference electrode.

### 3.1. Sensor with Pt Cermet Sensing Electrode

#### 3.1.1. Response Time, Drift, Reproducibility

For a typical sensor with Pt cermet sensing electrode, basic sensor characteristics, like response time, signal noise, drift and reproducibility, have been determined. In the temperature range 400–650 °C, the sensor responds to changes in oxygen concentration quickly. After a step change from 10 to 1000 ppm O_2_ in N_2_, Cell 1 shows a stable voltage reading after 7 s, while the sensor reaches 90% of the corresponding maximum signal amplitude after an additional 3–6 s. A different picture is observed at 350 °C, where even after 30 min the sensor voltage is still approaching a stable value. Voltage signal noise was determined under constant experimental conditions for periods of several days, yielding an average noise amplitude of 0.01–0.05 mV at 450–650 °C, which increased to a maximum of 0.54 mV at 350 °C. Over a period of 155 h at a constant temperature of 600 °C in N_2_, the sensor voltage drifted towards lower values by 0.12 mV. During a testing period of approximately 500 h, the sensor signal under equivalent conditions varied by no more than 1 mV without any discernible tendency. It should be noted that for both signal noise and drift, not only the sensor itself but also fluctuations of temperature, absolute and oxygen partial pressure and flow rate as well as signal acquisition, must be accounted for. Reproducibility was evaluated by comparing sensor voltages before and after an eight day measuring cycle, comprising multiple temperature changes in the range 350–650 °C, as well as varying oxygen concentration between 21 vol% and 13 ppm in N_2_. At the same temperature and oxygen concentration settings, voltages differed by 0.07 mV.

#### 3.1.2. Sensor Response in N_2_ and O_2_

**Figure 2:**
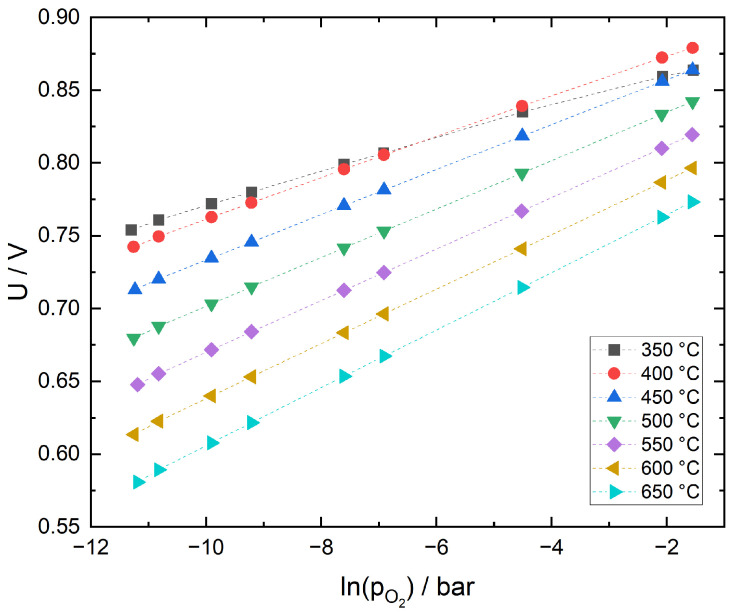
Sensor with Pt cermet sensing electrode, sensor voltage as function of oxygen partial pressure for different temperatures.

In Figure 2, the voltage of a sensor with Pt cermet sensing electrode is plotted as function of oxygen partial pressure for different sensor operating temperatures. For most of the temperature range, the data exhibits a logarithmic dependence on the oxygen partial pressure, indicating Nernstian behavior. However, at 350 °C, the plot shows a markedly lower slope of the data, causing them to intersect with the graphs for 400 and 450 °C.

One way to verify the proper function of the internal solid state reference electrode is by extracting the actual temperature from the measured sensor voltages. Applying linear fits, *T* was calculated from the slopes of the data using Equation (9),

(13) $$U=\frac{RT}{4F}\ln\frac{p_{O_{2}}^{ref}}{p_{O_{2}}^{s}}=\frac{\Delta G_{NiO}^{0}}{2F}-\frac{RT}{4F}\ln p_{O_{2}}^{s},$$

where indices ref and *s* indicate reference and sample gas. In Table 1, the calculated values are included as $T_{Nernst}$ and compared to the temperature $T_{TC}$ measured with the thermocouple in close proximity of the sensor. $|\Delta T_{Nernst}|$ is the standard deviation of the linear fit, while ΔT is the difference between $T_{Nernst}$ und $T_{TC}$.

Assuming that the measurement uncertainty of a type-S thermocouples is approximately 2–3 K, measured and calculated temperatures agree well in the range 450–650 °C. At 350 °C, neither the ionic conductivity of the 10Sc1CeSZ solid electrolyte nor the kinetics of the Pt cermet electrodes are limiting factors which could explain the large deviation between measured and calculated temperature. Both the long time needed to obtain a stable sensor voltage and the large ΔT suggest that the equilibrium reaction of the Ni/NiO solid state reference electrode is too slow to provide a sufficiently stable reference potential at this temperature.

For sensor operation, the reference oxygen partial pressure inside the sensor and possible deviations of the sensor voltage from theoretical values must be determined. For the couple Ni/NiO, various empirical formulae for $\Delta G^{0}$ can be found in the literature [9,42,43]. For the standard enthalpy of formation of NiO, Comert et al. have given a formula for the temperature range 760–1250 K (487–977 °C) [42]:

(14) $$\Delta G^{0}\left(NiO\right)=A+B\;\cdot \;T.$$

Here, *T* is the absolute temperature in K, while *A* and *B* are parameters obtained from experiment, yielding

(15) $$\Delta G^{0}\left(NiO\right)=-232,450+83.435\;\cdot \;T\;\cdot \;J\;\;\cdot \;\;{\mathrm{mol}}^{-1}.$$

Thus, for a given temperature, the reference oxygen partial pressure inside the cell can be determined both theoretically via Equation (9), as well as based on the measured sensor voltage *U*, via the Nernst equation

(16) $$p_{O_{2}}^{ref}=p_{O_{2}}^{s}\;\cdot \;\exp\frac{4F\;\cdot \;U}{RT}.$$

**Figure 3:**
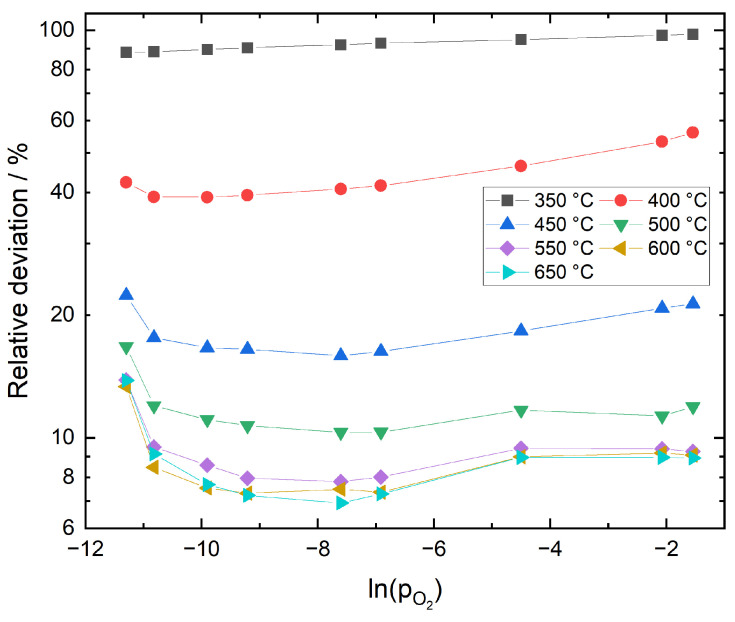
Relative deviation between theoretical reference oxygen partial pressures and values derived from experiment.

In Figure 3, relative deviations between theoretical reference oxygen partial pressures and values derived from experiment are plotted versus oxygen partial pressure in the sample gas for different temperatures. The data agree well for 550–650 °C but exhibit significant deviations for temperatures lower than 500 °C.

An obvious dependence of the deviations on oxygen partial pressure can be observed for all temperatures. It is important to note that experimental $p_{O_{2}}$ values are obtained by Cell 1, which is located in the feed gas line approximately 50 cm upstream of the sensor. Small deviations of the $p_{O_{2}}$ at the sensor location from the oxygen partial pressure at Cell 1 are expected due to dilution effects and minor leakages at low oxygen concentrations and the measurement uncertainty of the electrochemical electrolysis devices at high $p_{O_{2}}$. This is in good agreement with the fact that the highest deviations in Figure 3 are observed at both low and high oxygen partial pressures. However, the main reason for the deviations lies in the error caused by the temperature measurement. Based on a linearized error analysis for Equation (9), relative errors for calculated reference oxygen partial pressures have been determined for different temperatures and temperature errors. The values are summarized in Table 2.

They confirm the increase in deviation with decreasing temperature and show that even an error of 1 K during temperature measurement can result in the deviations observed in Figure 3. Furthermore, it should be stressed that the empirical formulae for $\Delta G^{0}\left(NiO\right)$ reported in literature are only based on experimental data for temperatures higher than 490 °C.

#### 3.1.3. Response Behavior in the Presence of CO

**Figure 4:**
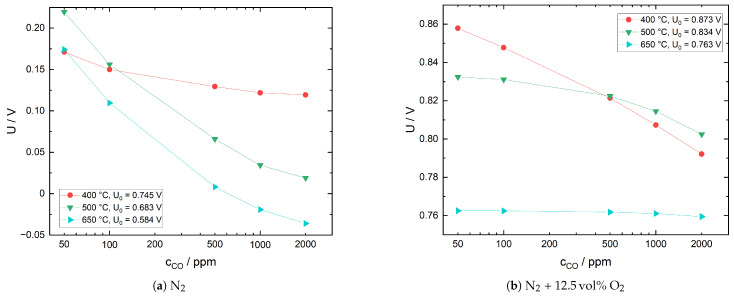
Sensor with Pt cermet sensing electrode, response behavior to CO in two different sample gas mixtures. Baseline sensor voltages in the absence of CO are given by $U_{0}$.

In Figure 4, the response behavior of the sensor with Pt cerment sensing electrode to various CO concentrations in both N_2_ and N_2_ with 12.5 vol% O_2_ is plotted. Baseline voltages $U_{0}$ are included for easier comparison. It should be noted that in the case of N_2_, the carrier gas contains 13 ppm of O_2_ due to gas line leakages. For N_2_, the plots show a decrease of the CO sensitivity with increasing CO concentration and an increase with increasing temperature. While the first observation also holds for N_2_ with 12.5 vol% O_2_, the CO sensitivity strongly decreases with increasing temperature in the presence of O_2_. This behavior can be explained based on the following theoretical considerations: Under the present measurement conditions, it is reasonable to assume that relevant solid-sample gas interactions are restricted to sensing electrode and solid electrolyte. Furthermore, additional measurements confirmed that gas phase CO oxidation in the quartz glass reactor is negligible at temperatures of up to 700 °C. Hence, CO interaction with the sensor is governed by two reactions: the catalytical oxidation at the Pt cermet electrode (Equation (11)) and the electrochemical oxidation at the triple phase boundary (Equation (12)). In the hypothetical case of complete chemical equilibration of CO and O_2_, the sensor voltage should correspond to the equilibrium oxygen partial pressure $p_{O_{2}}^{eq}$.

To evaluate to what extent the oxygen partial pressure at the sensing electrode deviates from $p_{O_{2}}^{eq}$, sensor voltages for $p_{O_{2}}^{eq}$ were calculated based on Equation (11). The equilibrium constant *K* of the reaction represented by Equation (11) is 10^11^–10^19^ in the relevant temperature range. Therefore, $p_{O_{2}}^{eq}$ will be very low for excess CO and $p_{CO}^{eq}$ will be low for excess oxygen. Assuming a carrier gas consisting of N_2_ with an initial oxygen concentration of 13 ppm due to line leakages, $p_{O_{2}}^{eq}$ has been estimated according to an approximation used by Brailsford et al. [44]. In the case of N_2_ with 12.5 vol% O_2_, complete CO conversion is assumed. While this is an extreme case, the influence on calculated sensor voltage in this concentration range is low.

**Figure 5:**
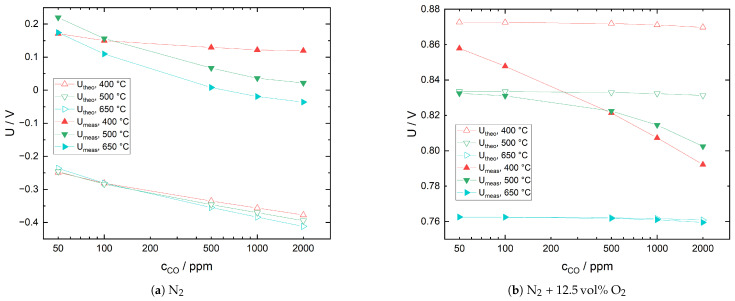
Sensor with Pt cermet sensing electrode under CO exposure in two different sample gas mixtures. Comparison of measured sensor voltages (U_meas) with theoretical values (U_theo) calculated under the assumption of complete equilibration of CO with available O_2_. All voltages are versus Ni/NiO reference electrode.

In Figure 5a,b the calculated sensor voltages are compared to measured values for three different temperatures. Theoretical sensor voltages in N_2_ are negative because the $p_{O_{2}}^{eq}$ are lower than the reference oxygen partial pressures at the corresponding temperature. At all temperatures, the difference between theoretical and measured values is approximately 400–500 mV. In N_2_ with 12.5 vol% O_2_, the differences are much smaller, with values of 15–78 mV at 400 °C and only 0.03–1.36 mV at 650 °C. In both gas environments, CO conversion increases with an increase in temperature, yet there appears to be no complete equilibration. However, in N_2_ with 12.5 vol% O_2_, the discrepancy between measured and equilibrium voltage is very small at 650 °C; therefore, it is assumed that the oxygen partial pressure in the sample gas, $p_{O_{2}}^{s}$, deviates only slightly from $p_{O_{2}}^{eq}$. Furthermore, at 500 and 650 °C, the sensor voltage exhibits an almost linear dependence on CO concentration. This behavior has been described by several authors with respect to the CO sensitivity of potentiometric solid electrolyte gas sensors under excess oxygen [45,46,47]. Garzon et al. argue that based on theoretical models, this dependency can be expected for mixed potentials close to equilibrium with an additional diffusion limitation of CO transport towards the triple phase boundary [47].

Based on the above results and additional adsorption/desorption studies on Pt powders previously reported in Reference [8], the following model for the sensor response is proposed:

##### a. N_2_

At 400 °C, the oxygen coverage of the sensing electrode surface is high as shown in Reference [8]; hence, a large fraction of incident CO is oxidized chemically. A small amount of CO, however, reaches the triple phase boundary and, along with the oxygen-reduction reaction (Equation (2)), contributes to the mixed potential. At 500 and 650 °C, electrode oxygen coverage is decreased significantly and catalytical CO oxidation only plays a minor role. Most of the CO reaches the triple phase boundary, where its electrochemical oxidation dominates the mixed potential due to the low oxygen concentration in the sample gas.

##### b. N_2_ with 12.5 vol% O_2_

When comparing to the measurements in N_2_, the CO sensitivity in N_2_ with 12.5 vol% O_2_ is considerably lower. This is attributed to two mechanisms: the higher oxygen coverage of the electrode surface, leading to an increased chemical conversion of CO, and the higher oxygen partial pressure at the triple phase boundary. Particularly at high temperatures, in the present studies at 650 °C, a large fraction of CO is oxidized on the electrode surface, therefore not contributing to the mixed potential. At this temperature, the gas mixture at the electrode is close to equilibrium. However, at 400 °C, the large deviation between measured and theoretical values suggests that despite excess oxygen, a significant share of CO passes the electrode surface without being oxidized and contributes to the mixed potential. The almost linear dependence of sensor voltage on CO concentration could be a sign of diffusion limited CO transport towards the sensing electrode.

### 3.2. Sensor with ITO Sensing Electrode

#### 3.2.1. Basic Sensor Characteristics in N_2_ and O_2_

**Figure 6:**
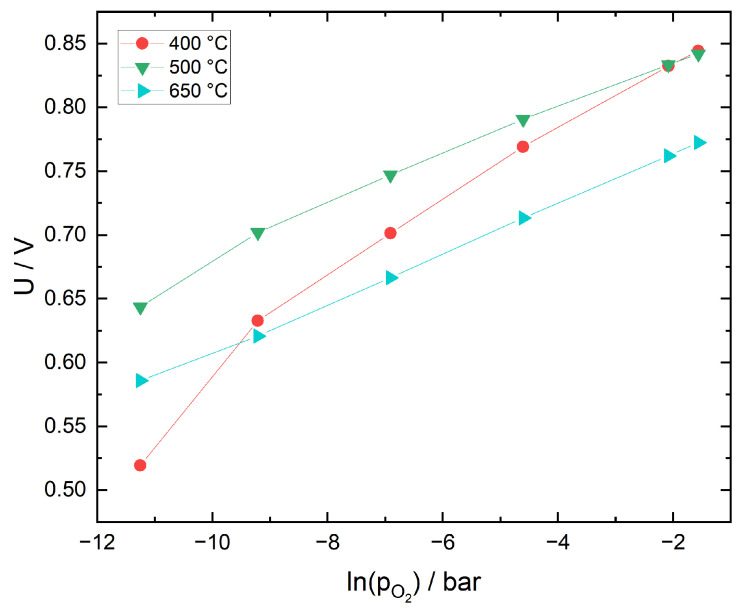
Sensor with indium tin oxide (ITO) sensing electrode, sensor voltage as function of oxygen partial pressure for different temperatures.

In Figure 6, the voltage response of the sensor with ITO sensing electrode is plotted as a function of oxygen partial pressure in N_2_ for different temperatures.

Only at 650 °C the data show a logarithmic dependence on oxygen partial pressure over the whole concentration range and thus, a Nernstian behavior. At 500 °C, the measured sensor voltage is lower than the equilibrium voltage only at low $p_{O_{2}}^{eq}$, at 400 °C over the whole range. This behavior is attributed to incomplete equilibration between oxygen molecules in the sample gas, oxygen vacancies in the solid electrolyte and electrons in the ITO electrode at the triple phase boundary. The temperature range where this effect is pronounced agrees well with the results of adsorption/desorption and electrical conductivity studies presented in Reference [40], indicating that oxygen adsorption is impaired by other surface species, like hydroxyl groups (OH^−^), carboxylate (CO_2_^−^) and carbonate groups (CO_3_^2−^) and other carbonaceous adsorbates here as well. As described in detail in Reference [40], some of these species are readily adsorbed or formed during ambient air exposure before measurements or from contaminations of the sample gas. In addition, the oxygen coverage of semiconducting metal oxide surfaces is also be restricted by electrostatic effects as pointed out by Kohl [48]. As oxygen adsorption and diffusion on the electrode surface close to the triple phase boundary play a crucial role in establishing electrochemical equilibrium, a limitation of these processes by other adsorbates will have a significant influence on potential generation.

#### 3.2.2. Response Behavior in the Presence of CO

**Figure 7:**
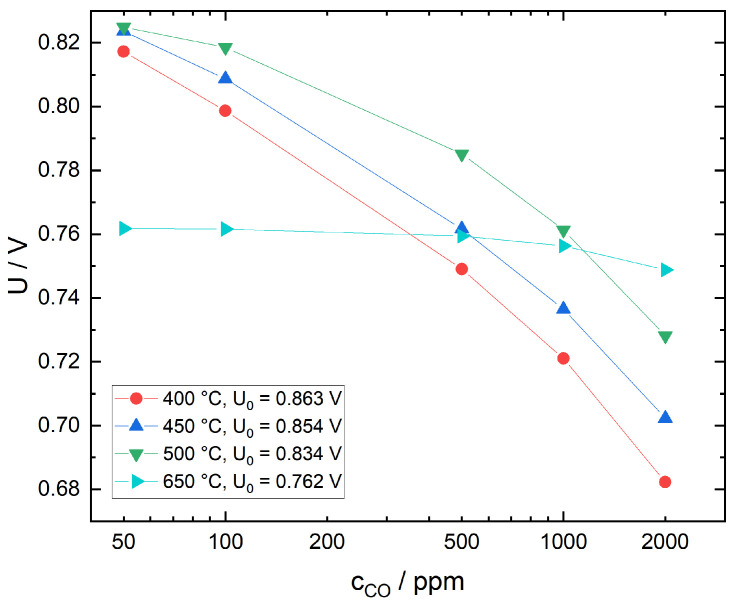
Sensor with ITO sensing electrode, sensor voltage as function of CO concentration for different temperatures in N_2_ with 12.5 vol% O_2_. Baseline sensor voltages in the absence of CO are given by $U_{0}$.

As the ITO sensing electrodes have been prepared in air, the investigation of the response behavior towards CO was limited to a base gas mixture of N_2_ with 12.5 vol% O_2_ to avoid the risk of excessive reduction of the electrode material and subsequent structural alteration. In Figure 7, the voltage response of a sensor with ITO sensing electrode is plotted as a function of CO concentration for different temperatures. Baseline voltages $U_{0}$ in N_2_ with 12.5 vol% O_2_ have been included for easier comparison. CO sensitivity at 650 °C is low but increases significantly with decreasing temperature. The observed behavior can be explained as follows: at low temperatures, the electrode surface is covered with various non-oxygen adsorbates as described in Section 3.2.1. This impairs oxygen adsorption, resulting in a decreased catalytic activity towards chemical CO oxidation. Part of the incident CO does in fact react with adsorbed oxygen to form carboxylates and carbonates. However, only a small fraction of these species decompose and desorb, leaving less active electrode area for subsequent O_2_ and CO interaction [40]. Meanwhile, a significant amount of CO reaches the triple phase boundary, where it is oxidized electrochemically, directly contributing to the mixed potential. At high temperatures, the kinetics of carboxylate and carbonate formation, decomposition and desorption as CO_2_ on the electrode surface is enhanced, leading to an increased chemical conversion of CO. As a consequence, less CO reaches the triple phase boundary so that the voltage measured corresponds closely to the equilibrium oxygen partial pressure.

### 3.3. Comparison of Sensor with Pt Cermet vs. ITO Sensing Electrode

#### 3.3.1. Sensor Voltage as a Function of Oxygen Partial Pressure

**Figure 8:**
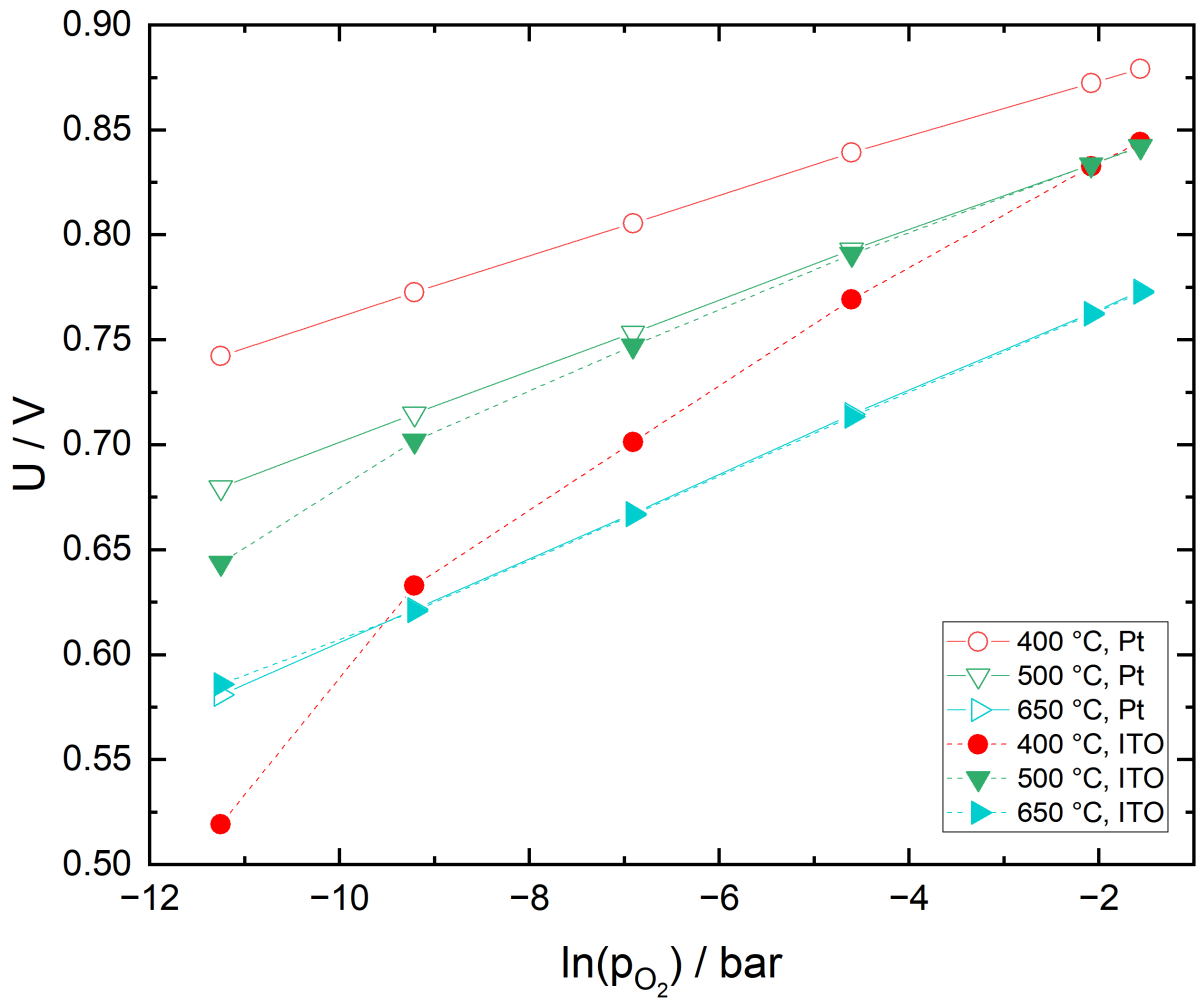
Comparison of sensor voltages for a sensor with Pt cermet versus ITO sensing electrode as function of oxygen partial pressure in N_2_ for different temperatures.

In Figure 8, sensor voltages as a function of oxygen partial pressure are compared for Pt cermet and ITO sensing electrodes at selected temperatures. While the sensor with Pt cermet SE exhibits a logarithmic dependence on oxygen partial pressure at all temperatures, the response of the ITO SE significantly deviates at 400 and 500 °C, with an offset of 35 mV at 21 vol% O_2_ and 400 °C. The reason for this behavior has been explained in the previous section. Although the behavior of the sensor with ITO SE at low temperatures is sub-Nernstian, its sensitivity to changes of the oxygen partial pressure is significantly higher than with a Pt cermet SE.

#### 3.3.2. Sensor Response to CO

**Figure 9:**
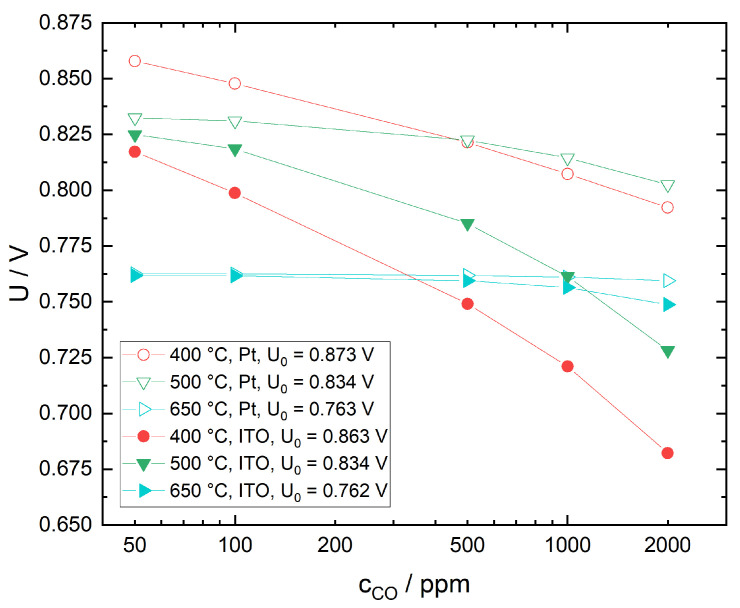
Comparison of sensor voltages for a sensor with Pt cermet versus ITO sensing electrode as a function of CO concentration in N_2_ with 12.5 vol% O_2_ for different temperatures. Baseline sensor voltages in the absence of CO are given by $U_{0}$.

In Figure 9, sensor response to CO in N_2_ with 12.5 vol% O_2_ is compared for Pt cermet versus ITO sensing electrode. While the CO sensitivity of both electrodes differs only slightly at 650 °C, at 400 °C the CO sensitivity of the ITO SE is higher than the Pt cermet SE by a factor of 2–3. This is in very good agreement with the lower catalytic activity towards CO oxidation observed for ITO in CO chemisorption studies reported in a previous article [40]. The very similar behavior at 650 °C is an indication, that the kinetics at the ITO/10Sc1CeSZ interface is not significantly limited in comparison to Pt/10Sc1CeSZ.

## 4. Conclusions

When deployed in zirconia-based potentiometric solid electrolyte gas sensors, indium tin oxide sensing electrodes may show Nernstian behavior towards oxygen in inert gas at high temperatures. At 650 °C, for example, significant deviations from the voltage response of platinum cermet electrodes are only observed at very low oxygen partial pressures. At low temperatures, particularly below 500 °C, equilibration at the triple phase boundary is constricted, leading to a considerably higher sensitivity towards oxygen as compared to platinum cermet electrodes. This behavior can be explained by the presence of contaminations on the electrode surface, leading to a reduced oxygen adsorption and diffusion. These species are readily adsorbed or formed during ambient air exposure and from contaminations of the sample gas. Among these are various carbonaceous species, which are also formed on the electrode surface during exposure to carbon monoxide. The incident reducing gas reacts with oxygen adsorbed at the surface, forming carboxylates and carbonates. At high temperatures, these product species quickly decompose and desorb, thus enhancing chemical carbon monoxide conversion. As a result, only a small fraction of carbon monoxide reaches the triple phase boundary and contributes to the mixed potential. Carbon monoxide sensitivity is low and measured voltages deviate only slightly from those obtained with platinum cermet electrodes. At low temperatures, decomposition and desorption of carboxylate and carbonate species on the ITO surface proceed very slowly, effectively limiting the electrode surface available for further oxygen and carbon monoxide interaction. The result is a reduced chemical conversion, allowing more carbon monoxide to reach the triple phase boundary. Its contribution to the mixed potential is enhanced, leading to a significantly higher sensitivity as compared to platinum cermet electrodes.

## Figures and Tables

**Table 1:** Comparison of temperatures obtained from thermocouple measurement in close proximity to the sensor with values derived from sensor voltage.

$T_{\mathrm{TC}}$	$T_{\mathrm{Nernst}}$	$|\Delta T_{\mathrm{Nernst}}|$	|ΔT|
°C	°C	°C	°C
350.8	249.6	7.2	101.1
400.2	379.2	3.7	21.0
450.1	447.8	2.1	2.3
500.4	501.4	1.8	1.0
549.8	550.6	1.7	0.8
599.0	599.4	1.9	0.5
648.9	649.6	2.2	0.8

**Table 2:** Theoretical reference oxygen partial pressure values $p_{O_{2}}^{ref,theo}$ calculated for different temperatures and resulting relative errors due to an assumed uncertainty in temperature measurement, given by ΔT.

T	ΔT	$p_{O_{2}}^{\mathrm{ref},\mathrm{theo}}$	Rel. Error
°C	K	bar	%
350	1	$5.59\times 10^{-31}$	14
350	5	$5.59\times 10^{-31}$	72
500	1	$2.03\times 10^{-23}$	9
500	5	$2.03\times 10^{-23}$	47
650	1	$2.58\times 10^{-18}$	7
650	5	$2.58\times 10^{-18}$	33

## Data Availability

The data presented in this study are available on request from the corresponding author.

## Abbreviations

The following abbreviations are used in this manuscript:

8YSZ	Zirconia, doped with 8 mole % Y_2_O_3_
10Sc1CeSZ	Zirconia, doped with 10 mole % Sc_2_O_3_ and 1 mole % CeO_2_
ITO	Indium tin oxide
M	Metal
RE	Reference electrode
SE	Sensing electrode
SZ	Stabilized zirconia
TC	Thermocouple
